# Design and dosimetry characteristics of a commercial applicator system for intra‐operative electron beam therapy utilizing ELEKTA Precise accelerator

**DOI:** 10.1120/jacmp.v11i4.3244

**Published:** 2010-07-19

**Authors:** Alexander Nevelsky, Zvi Bernstein, Raquel Bar‐Deroma, Abraham Kuten, Itzhak Orion

**Affiliations:** ^1^ Department of Oncology Rambam Medical Center Haifa Israel; ^2^ Department of Nuclear Engineering Ben‐Gurion University of the Negev Beer‐Sheva Israel

**Keywords:** intraoperative radiotherapy, electron dosimetry, linear accelerators

## Abstract

The design concept and dosimetric characteristics of a new applicator system for intraoperative radiation therapy (IORT) are presented in this work. A new hard‐docking commercial system includes polymethylmethacrylate (PMMA) applicators with different diameters and applicator end angles and a set of secondary lead collimators. A telescopic device allows changing of source‐to‐surface distance (SSD). All measurements were performed for 6, 9, 12 and 18 MeV electron energies. Output factors and percentage depth doses (PDD) were measured in a water phantom using a plane‐parallel ion chamber. Isodose contours and radiation leakage were measured using a solid water phantom and radiographic films. The dependence of PDD on SSD was checked for the applicators with the smallest and the biggest diameters. SSD dependence of the output factors was measured. Hardcopies of PDD and isodose contours were prepared to help the team during the procedure on deciding applicator size and energy to be chosen. Applicator output factors are a function of energy, applicator size and applicator type. Dependence of SSD correction factors on applicator size and applicator type was found to be weak. The same SSD correction will be applied for all applicators in use for each energy. The radiation leakage through the applicators is clinically acceptable. The applicator system enables effective collimation of electron beams for IORT. The data presented are sufficient for applicator, energy and monitor unit selection for IORT treatment of a patient.

PACS number: 87.00.00, 29.20.‐c

## I. INTRODUCTION

During the last few years, intraoperative radiotherapy (IORT) with electron beams has been reintroduced into the clinic (primarily due to mobile linear accelerators). Intraoperative radiotherapy involves the delivery of a single large radiation dose to the exposed tumor at the time of surgical exploration or to the bed of a resected tumor. When a conventional, non‐dedicated accelerator is employed for intraoperative radiotherapy, a specially designed IORT applicator system must be used.

An applicator system for IORT serves three major functions: collimation of the electron beam, delineation of treatment volume, and retraction of normal tissues. The applicator used at our institution is a commercial device of the hard‐docking type, which means that the treatment applicator positioned in the patient is rigidly attached to the collimator head of the linear accelerator. Some of the IORT systems of the hard‐docking type have been previously described in the literature.^(^ ^1^ ^–^ ^4^ ^)^ The recent publications on the dosimetry and design of a soft‐docking type IORT device are also important.^(^ ^5^ ^,^ ^6^ ^)^ To the best of our knowledge, no data on the IORT applicator used at our institution has been published.

The aim of this work is to present the data that we deemed necessary for clinical use of the IORT device. The lateral extent and depth of the target volume can be determined by direct manipulation in the surgical wound or by means of an ultrasonic scan with the transducer inserted into the surgical cavity. Energy and applicator size are then selected such that the 90% isodose surface, to which the radiation oncologist prescribes dose, would cover this target volume with limited margins. The depth dose data and dose distributions should be presented in a graphical way that makes the task of applicator and energy selection fast and comprehensive. Dosimetric measurements were made to allow calculation of the MU setting for any irradiation condition (energy, applicator size, treatment distance). From these data, the delivered dose, D, and monitor units, MU, are related by the equation (assumingthat1MU=1cGy):

(1) D=MU×PDD(d)/100×OF×S

In this equation, PDD(d) is the percent depth‐dose (along the clinical central axis, defined later), OF is the applicator output factor (dose per monitor unit at the depth of maximal dose and at the SSD=100cm), S is the SSD correction factor.

It was also recommended that, before placing an IORT system into clinical service, extent of radiation leakage through the applicator wall should be determined.^(^ ^7^ ^)^

## II. MATERIALS AND METHODS

### A. IORT device description

**Figure 1:**
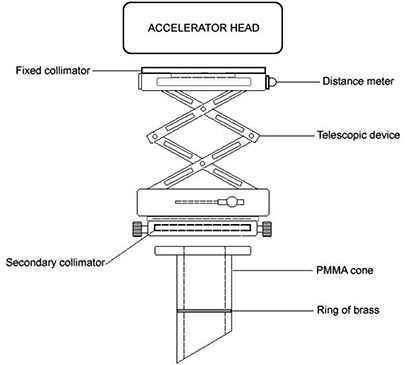
Illustration of the main components of IORT device.

**Figure 2:**
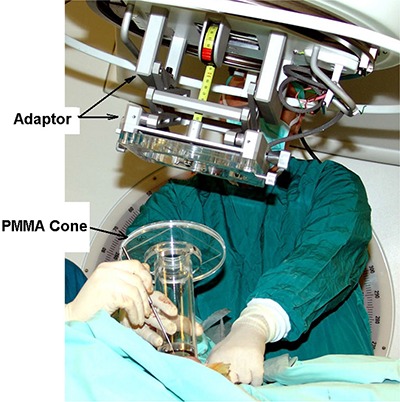
Photograph of the IORT device.

The IORT device manufactured by Arplay Medical (Izeure, France) was developed in collaboration with the Lyon Sud hospital. For IORT treatments at our institution, the device is attached to an Elekta Precise (Elekta Oncology Systems) accelerator. The device includes an adapter that connects to the linac head, and a set of PMMA applicators with circular cross sections. The major components of the system are schematically shown in Fig. 1. A photograph of the system is shown in Fig. 2.

The telescopic device allows changing of the source‐to‐ applicator‐end distance in the range up to 100–110 cm. This makes the task of positioning a patient and attachment of the applicator to the adapter comfortable.

The fixed collimator has a square aperture of 10 by $10\;{\text{cm}}^{2}$. The secondary collimators are made of lead (thickness of 0.5 cm) and have circular apertures. The secondary collimator aperture size is 0.2 cm less than the inner diameter of the applicator in use. The shape of the fixed collimator (square), and the thickness and dimensions of the lead collimator are defined by the manufacturer. Collimator jaws settings of the accelerator are kept constant at 14 by 14 cm.

A total of 10 circular applicators of different diameters and end angles made of PMMA with a wall thickness of 0.4 cm are available. The applicators inner diameters are 40, 50, 60, 80 and 100 mm. The applicator end, which is positioned in the patient, can be straight or beveled to 30°. At the patient end of the applicator, a ring of brass is placed to minimize the dose contribution from the wall scatter. PMMA offers the advantage of being transparent compared to metallic applicators, which makes positioning of the applicator comfortable for the surgeon.

After the applicator is positioned in the surgical wound, the patient is brought to the accelerator on a surgical couch that can be tilted in the longitudinal and lateral directions, and can be moved up and down. By changing height and tilt of the couch as well as gantry angle and source‐to‐ applicator‐end distance, the upper part of the applicator is inserted into the IORT device adapter and secured in this position with two nuts. Distance meter readings, which are transformed into SSD, are taken at this stage.

### B. Depth‐dose measurements

**Figure 3:**
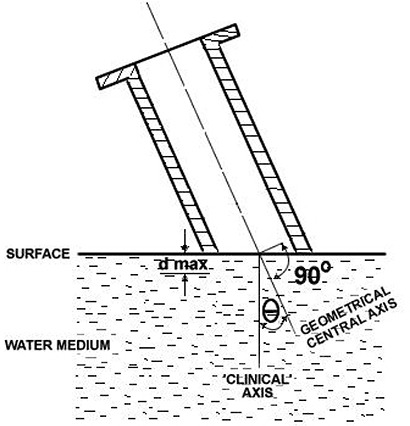
Cross‐sectional view of the IORT positioned above a phantom with definition of the geometrical and clinical axes.

The central axis for beveled applicators has little significance clinically because vertical depth from the phantom surface has more relevance for IORT. Therefore, a new “clinical” axis is defined as the line projecting perpendicularly from the phantom surface and intersecting the central axis of the applicator at the surface, as shown in Fig. 3.^(^ ^8^ ^)^

For straight applicators, the angle of incidence Θ is equal to 0°. However, for beveled applicators, Θ is equal to the angle of the bevel, and the clinical and geometrical central axes are not identical.

The depth doses for all applicators were measured in a water phantom along the clinical axis and normalized such that 100% represents the value of maximum dose. Beveled applicators were placed with the beveled end along the cross‐plane direction (i.e. flush with the water surface).

All scanning and point measurements were made in water using a PTW Markus ion chamber (PTW FreiburgGmbH, Freiburg, Germany) with a commercially available water phantom (The Blue Phantom, IBA Dosimetry). Depth‐ionization curves were converted into depth‐dose curves using the available AAPM protocols based on the TG‐25 report.^(^ ^9^ ^)^

All measurements were performed at SSD=100cm. For the smallest and the biggest applicators, depth doses were also measured at SSD=110cm. The differences in depth dose were found to be negligible in both cases. Hence, depth dose changes as a result of SSD changes will be ignored in the calculations.

### C. Output factors

Applicator output factors are defined as the ratio of output for the applicator of interest to the output for the $14\times 14{\text{cm}}^{2}$ standard electron applicator. First, electrometer readings (Mref), were taken in reference conditions $(14\times 14{\text{cm}}^{2}$ applicator with SSD=100cm) for a fixed number of monitor units, for all energies in use. The output factors were then measured by placing the ion chamber at $d_{\max}$ along the clinical axis. At $d_{\max}$ position, the electrometer reading Miort was taken for the same number of monitor units as it was in the measurement of Mref. Although there might be some differences of the stopping‐power ratio and the perturbation factor between the two irradiation setups, these differences are sufficiently small to have little effect on the ionization to dose conversion. This additional uncertainty in output was estimated to be between 1% and 2%.^(^ ^6^ ^)^

Hence, the signal from the ion chamber, M, can be considered directly proportional to the absorbed dose to water, D. Thus, for the same number of monitor units, the following relationship is valid at $d_{\max}$:

(2) Diort=(Miort/Mref)*Dref    [Gy]

The accelerator is calibrated to deliver 1 cGy per MU at $d_{\max}$ for all nominal electron energies in reference conditions. Hence, the output factor OF in the IORT geometry and at $d_{\max}$ is given by:

(3) OF=Miort/Mref   [cGy/MU]

Repeated measurements using the ion chamber were made under reference conditions at the beginning and at the end of the measurement series, to correct for any changes in accelerator output.

Applicator output factors were measured for all 40 energy/applicator combinations.

### D. SSD correction

For the SSD correction factor, we decided to rely on the interpolated results of measurements at three different distances: SSD=100cm,SSD=105cm and SSD=110cm. Measurements were performed using a Markus chamber placed in the RMI 457 Solid Water phantom (Gammex Inc., Middleton, WI) and Farmer 2570 electrometer (NE Technology Ltd., Berkshire, UK). An SSD correction factor S was defined as the ratio of output at arbitrary SSD to the output at SSD 100 cm for the same applicator and the same energy. Thus, SSD correction factor for SSD 105 cm and SSD 110 cm could be found as the ratio of electrometer readings M at the corresponding distances to the electrometer reading M (100) at SSD 100 cm.

$$\begin{array}{l} S(100\;\text{cm})=1\\ S(105\;\text{cm})=M(105)/M(100)\\ S(110\;\text{cm})=M(110)/M(100) \end{array}$$

Based on these three points, a quadratic polynomial was created in order to allow calculation of SSD factor for any SSD between 100 cm and 110 cm.

(4) $$S=A_{1}*\text{SSD}{\left(\text{cm}\right)}^{2}+A_{2}*\text{SSD}(\text{cm})+A_{3}$$

### E. Isodose measurement

KODAK X‐OMAT (Eastman Kodak, Rochester, NY) ready‐packed films were sandwiched between slabs of Solid Water RMI 457 phantom and irradiated edge‐on in the IORT set‐up at SSD=100cm. For consistency, each film was irradiated in the cross‐plane direction, which was required for beveled applicator measurements made in the plane containing the applicator's major elliptical axis.

Film optical density was subsequently analyzed using a VIDAR 16 Pro film scanner and the Wellhofer OmniPro software (IBA Dosimetry America, Bartlett, TN, U.S.A.). Isodose contours for all energy/applicator combinations were produced.

### F. Radiation leakage measurements

One of the advantages of IORT is that the area to be irradiated can be localized and critical normal tissue can be placed outside the target area. Thus normal tissue will receive much lower dose.

However, the dose to the surrounding tissue can still be greater than desired due to the leakage through the PMMA applicator wall and through the collimation system. The tissue of concern may be located lateral to the treated area beyond the applicator end or next to the side of the applicator.

The effect of leakage was measured by placing X‐OMAT radiographic film in a solid water phantom perpendicular to the outer surface of the applicator and parallel to the central axis. Comparison of this exposed film to one exposed at the end of the IORT applicator demonstrated the amount of leakage and where it is greatest.

## III. RESULTS

**Figure 4:**
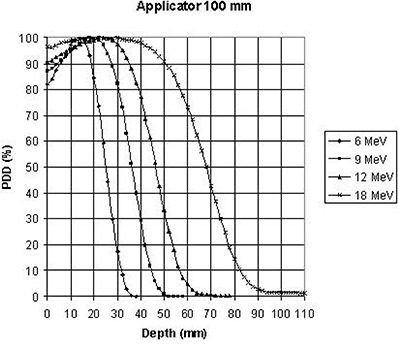
Straight end IORT device: measured PDD along the clinical axis for each beam energy.

**Figure 5:**
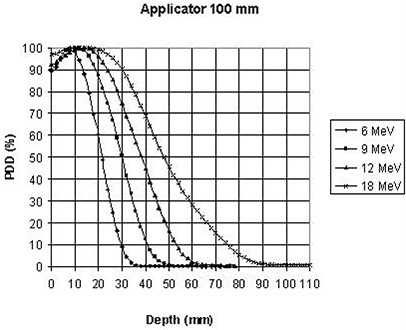
Beveled end IORT device: measured PDD along the clinical axis for each beam energy.

The percent depth doses along the clinical axis of the largest applicator (100 mm) as a function of energy are plotted for the straight and beveled end, respectively, in Fig. 4 and Fig. 5.

These plots illustrate the fact that for all measured applicator sizes and energies, depth doses for the beveled applicators were lower than depth doses for the straight applicators for the same respective applicator diameter and energy. This difference should be taken into account during energy selection for an IORT treatment.

**Figure 6:**
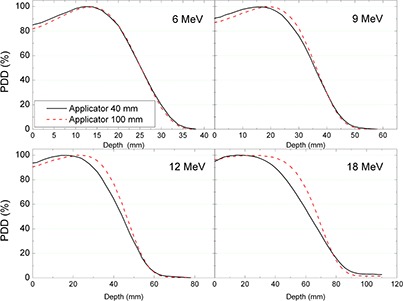
Graphical comparison of measured PDD for 40 mm and for 100 mm applicators, straight end in different energies.

Graphical comparison of measured depth doses for the largest (100 mm) and the smallest (40 mm) applicators is presented in Fig. 6. As expected, the difference in PDDs becomes more pronounced as the beam energy increases.

### A. Output

**Figure 7:**
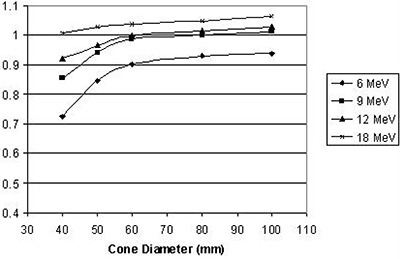
Output factors for straight applicators versus applicator diameter for each beam energy.

**Figure 8:**
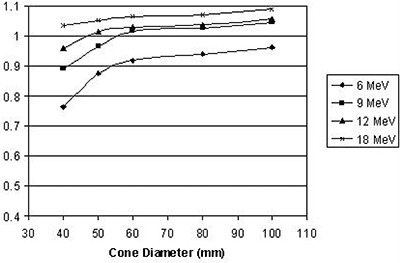
Output factors for beveled applicators versus applicator diameter for each beam energy.

Output factors as a function of applicator diameter and electron energy are presented for the straight and the beveled applicators in Fig. 7 and Fig. 8, respectively.

It can be seen that, for the IORT applicator, the output dependence on field size is stronger, especially for low energies. The reason for that variation is the difference in design of the standard and the IORT applicators, specifically the position of the final collimator. In the standard applicator, the field is defined by an insert which is placed very close (usually 5 cm) to the skin. In the IORT applicator, field size is defined by the secondary collimator (see Fig. 1) which is situated 25 cm from the applicator end. The concept of side‐scatter equilibrium is very important in understanding the variation of output as function of collimator size and position. At the level of a final beam‐defining collimator, if more than 99% of electrons that could reach the point of interest (measurement) in the phantom pass through the collimator opening, then side‐scatter equilibrium is said to exist. When the collimator closes sufficiently, some electrons that could have reached the point of interest are removed from the beam and side‐scatter equilibrium is lost, and this leads to output factor decrease. Obviously, when the collimator is placed further from the point of interest, the field size at which the loss of side‐scatter equilibrium takes place increases. This effect is most significant at low electron energies, since the angular scattering power for electrons is approximately inversely proportional to the square of the energy. (See Ref. 2)

### B. SSD correction

SSD correction factors were found to be applicator size independent (differences less than 0.4%). This observation contradicts the general understanding with external electron beams which exhibit strong field size dependence. The reason for this contradiction is again in IORT applicator design: when SSD changes, the secondary collimator which defines the radiation field moves further from the source so that SSD variations do not lead to changes in the field size. SSD correction factors averaged over all applicators sizes are presented in Table 1 and Table 2, for the straight and beveled applicators, respectively.

**Table 1:** SSD correction factors for the straight applicators.

*Energy/SSD*	*100*	*105*	*110*
6 MeV	1.000	0.875	0.768
9 MeV	1.000	0.891	0.797
12 MeV	1.000	0.897	0.809
18 MeV	1.000	0.900	0.817

**Table 2:** SSD correction factors for the beveled applicators.

*Energyl/SSD*	*100*	*105*	*110*
6 MeV	1.000	0.886	0.780
9 MeV	1.000	0.893	0.803
12 MeV	1.000	0.898	0.805
18 MeV	1.000	0.898	0.814

It can be seen that the maximum difference in the SSD correction factors between the straight and the beveled end applicators is 1.5%, for the 6 MeV energy and SSD 110 cm. These differences were deemed clinically insignificant, and it was decided to apply the same SSD correction factors for all applicators in use. For this purpose, the average values of the SSD factors in Tables 1 and 2 were taken (Table 3). Coefficients of the quadratic polynomials are presented in Table 4.

**Table 3:** SSD correction factors for all applicator sizes and types.

*Energy/SSD*	*100*	*105*	*110*
6 MeV	1.000	0.880	0.774
9 MeV	1.000	0.892	0.800
12 MeV	1.000	0.898	0.807
18 MeV	1.000	0.899	0.815

**Table 4:** Coefficients of the quadratic polynomials for calculation of SSD correction factors.

*Energy*	$A_{1}$	$A_{2}$	$A_{3}$	$R^{2}$
6 MeV	0.000262	‐0.077646	6.143542	1.000
9 MeV	0.000304	‐0.083923	6.348482	1.000
12 MeV	0.000239	‐0.069577	5.563333	1.000
18 MeV	0.000351	‐0.092150	6.706427	1.000

### C. Isodoses

**Figure 9:**
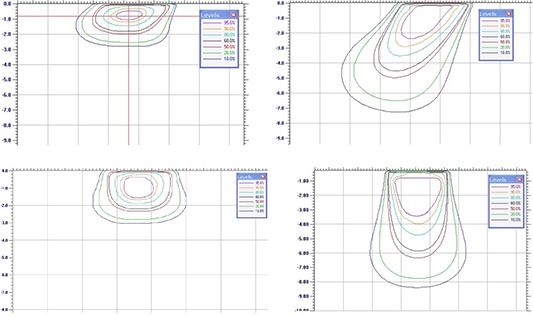
Isodose contours for beveled ((a),(b)) and for straight ((c),(d)) applicators for the 6 MeV and 18 MeV beams for the 40 mm applicators.

**Figure 10:**
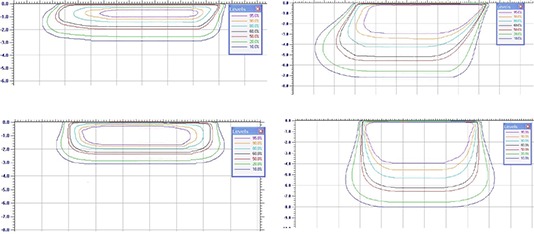
Isodose contours for beveled ((a),(b)) and for straight ((c),(d)) applicators for the 6 MeV and 18 MeV beams for the 100 mm applicators.

Isodose contours for beveled and for straight applicators for the 6 MeV and 18 MeV beams have been plotted for the 40 mm and 100 mm applicators in Fig. 9 and Fig. 10, respectively. Straight applicators produce a circular field and, therefore, the isodose distributions of Figs. 9 (c) and (d) and 10 (c) and (d) represent any plane coincident with the clinical axis. For beveled applicators, the isodose distributions of Figs. 9 (a) and (b) and 10 (a) and (b) represent the plane containing the applicator's major elliptical axis, and are asymmetrical about the clinical axis. The beveled applicator isodose distributions for the plane containing the applicator's minor elliptical axis are symmetrical about the clinical axis, and are closely represented by the straight‐applicator distributions for the same respective applicator diameter and energy. The rounding of the isodose lines for the higher electron energies can be observed, as well as the difference between straight and beveled applicators. These results emphasize that the radiation oncologist and the surgeon must be aware of the difference in the shape of the isodose distributions when the beveled applicators are used. Depth dose data on the central axis are not enough for selection of energy and applicator size for adequate coverage of the target.

### D. Leakage

**Figure 11:**
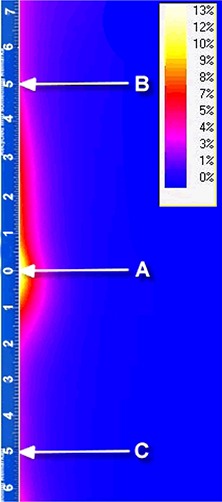
Leakage through the applicator wall. Distribution of absorbed dose along the wall of the 100 mm applicator at 18 Mev: A ‐ at the vicinity of the brass ring; B ‐ at 5 cm above the brass ring; C ‐ at 5 cm below the brass ring. Blue=<2%; pink=3%‐4%; red=5%‐8%; yellow=9%‐13%.

The collimation system of the IORT device allowed for minimal leakage to the patient. Radiation leakage through the applicator increased with applicator size and beam energy, and was found to be greatest for the 100 mm applicator and 18 MeV beam. Distribution of absorbed dose along the wall of the 100 mm applicator at 18 Mev is shown in Fig. 11. Note that the maximum dose was at the applicator surface in the vicinity of the brass ring (point A). At this point, the radiation leakage was about 13% of the maximum dose to the target. At point B (5 cm above the brass ring), the leakage was about 8% and, at the point C (5 cm below the brass ring), the leakage was about 3%. For the 40 mm applicator and 18 MeV beam, the doses at points A, B and C were 5%, 4% and 2% respectively. Radiation leakage for the beams with lower energy significantly decreased.

## IV. CONCLUSIONS

Applicator output factors were a function of energy, applicator size and applicator type. The overall range of output factors was demonstrated to be 0.726–1.089 for all applicator sizes and energies. Properties of output factors were qualitatively explained by a complex dependence of scattered radiation on geometry and on energy.

Analyzing IORT depth dose data for beveled applicators required definition of “clinical” central axes. PDD values for beveled applicators were less than the corresponding percent depth doses for the straight applicators.

Dependence of SSD correction factors on applicator size and applicator type was found to be weak. For each energy, the same SSD corrections were applied for all applicators in use. Calculation of SSD correction factors were based on the quadratic polynomial equations valid for all SSD values between 100 cm and 110 cm.

Examples of isodose distribution were presented. These examples demonstrated how the geometric coverage of the 90% isodose contour varied as a function of electron energy, applicator size and applicator type.

We have found that the prescription of the electron beam energy should be based on the isodose distribution rather on depth dose data on the central axis alone.

The radiation leakage through the applicators is clinically acceptable (maximum about 13%; practically less than 8% for all applicators and energies).

The data presented above are sufficient for applicator, energy and monitor unit selection for IORT treatment of a patient.
